# Medium-Term Outcomes of Stent Therapy for Aortic Coarctation in Children Under 30 kg with New Generation Low-Profile Stents: A Follow-Up Study of a Single Centre Experience

**DOI:** 10.1007/s00246-023-03402-8

**Published:** 2024-02-05

**Authors:** Jack J. C. Gibb, Wan Cheol Kim, Francisco Gonzalez Barlatay, Andrew Tometzki, Alan Pateman, Massimo Caputo, Demetris Taliotis

**Affiliations:** 1 Bristol Royal Hospital for Children, University Hospitals Bristol and Weston NHS Foundation Trust, Paul O’Gorman Building, Upper Maudlin Street, Bristol, BS2 8BJ UK; 2 Division of Cardiology, Department of Medicine, Dalhousie University, Halifax, NS B3H 3A7 Canada; 3 Noah’s Ark Children’s Hospital for Wales, Heath Park, Cardiff, CF14 4XW UK

**Keywords:** Paediatric interventions, Coarctation of aorta, Stent, Retrospective study

## Abstract

We previously reported short-term outcomes for stenting of aortic coarctation (CoA) (native or re-coarctation) with newer generation low-profile stents (Valeo, Formula, and Begraft stents) in children under 30 kg. We present here the medium-term outcomes of this procedure. Retrospective review of patients weighing under 30 kg who had percutaneous stent treatments for coarctation between 2012 and 2021 was performed. Clinical and procedural data were collected; 19 patients were included. The median age at the time of procedure was 5.1 [4.1–6.4] years and median weight 21.0 [17.3–22.3] kg. One patient had a history of re-coarctation. Thirteen (68%) patients were on anti-hypertensives pre-procedure. Different types of stents were used (14 Valeo™, 4 Formula^®^ 535, 1 BeGraft), which can all be dilated to 18 mm or larger. One patient required a 9 F sheath, all others required a 7 F sheath. The narrowest diameter in the aorta increased from a median of 3.5 [3.0–4.5] to 9.4 [8.9–9.8] mm, *p* < 0.001; there was a reduction in the median pressure gradient across the coarctation from 35.0 [30.0–43.0] to 5.0 [0–10.0] mmHg, *p* < 0.001. There were no intra-procedural complications. Follow-up was for a median of 56.0 [13.0–65.0] months. Five (26%) of patients underwent re-intervention after a median time frame of 40.0 [39.5–52.0] months; four had balloon dilation, one had repeat stent implantation. Five (26%) patients were on anti-hypertensive agent(s) post-intervention. Our single centre experience demonstrates that percutaneous stenting for coarctation of aorta in children under 30 kg, with low-profile stents, had no significant complications during the median follow-up time of 56 months. This study demonstrated that the procedure is safe and effective for short and medium-term therapy in this group of patients with a 26% re-intervention rate. A quarter of patients remained on anti-hypertensive medication post stenting, emphasizing the importance of long-term follow-up.

## Introduction

Aortic coarctation (CoA) has an overall incidence of 5/10,000 live births and, if left untreated, can lead to significant morbidity and mortality [1, 2]. Compared to balloon angioplasty alone, stent implantation for CoA provides superior dilation and avoids acute re-modelling of the aortic wall [3–5]. Stent delivery is limited by femoral artery size in small children. The COAST trial demonstrated the feasibility and safety of coarctation stenting in a large prospective multicentre study, however, all patients in this study were over 30 kg [6]. We previously reported our short-term outcomes of CoA treatment using Valeo™ (BD, NJ, USA) stents in children weighing under 30 kg, which can be dilated to adult size (18 mm) [7]. We now report the medium-term outcomes of CoA stenting in our centre. We aimed to report the need for ongoing anti-hypertensive therapy, re-intervention and peri-procedural, short and medium-term complications related to the procedure.

## Methods

As described previously, all patients weighing under 30 kg with a diagnosis of coarctation of the aorta, who underwent stent implantation in our centre, were identified from the departmental cardiology database (HeartSuite™) [7]. Patient demographics and clinical data were collected from the medical notes and HeartSuite™. Angiographic and haemodynamic data, including the diameter of the coarctation and pressure gradients before and after stent implantation were collated from catheterisation reports and angiograms and complications assessed using the clinical notes and electronic records. Vascular complications included vascular occlusion, arteriovenous fistulae or pseudo-aneurysms, identified on ultrasound if there had been clinical suspicion of vascular injury. Cerebral complications included cerebral infarction or bleeding, based on clinical suspicion or confirmed by brain MRI or computer tomography (CT). We also assessed for evidence of aortic dissection, aneurysm or pseudoaneurysm formation, stent fracture, deformation or migration or neo-intimal proliferation on CT and angiography for those requiring re-intervention.

Data are expressed as medians with interquartile ranges [IQR] and percentages as appropriate. Comparisons before and after stent implantation were performed using the paired Student’s *t*-test and a *p* value <0.05 was considered significant. All statistical analyses were performed using IBM^®^ SPSS^®^ Statistics Version 27 (IBM, Armonk, New York).

### Stent Descriptions

The Valeo stent (BD, NJ, USA) is a bare metal stent that has a triple helix design laser cut from a 316-L stainless steel tube. The stent is pre-mounted on a low-profile nylon balloon accepting a 0.035-in. guide wire and accommodating inflation pressures up to 14 atm. The stent is available in diameters between 6 and 10 mm and lengths between 18 and 56 mm. The required sheath size is 6 F for stents mounted on 6–8 mm balloons and 7 F for the larger stents mounted on 9–10 mm balloons. The open cell architecture of the stent allows it to be post dilated with minimal shortening [8, 9]. The 6–8 mm Valeo stents can be maximally dilated to 13 mm before fracturing. In our case series, only the 9 and 10 mm Valeo stents are used to take advantage of its maximal expandability to 20 mm.

The Formula 535 Vascular Stent (Cook, IN, USA) is a bare metal stent that has a slotted tube configuration made of 316L stainless steel. The stent is pre-mounted on a balloon catheter and deployed over a 0.035 inch guidewire with nominal and burst pressures of 8 and 10 atm, respectively. The stent is available in diameters between 4 and 10 mm and lengths between 12 and 60 mm. The sheath size is 7 F for 9–10 mm diameters stents, 6 F for 5–8 mm diameter stents, and 5 F for 4 mm diameter stents. The 10 and 9 mm diameter stent is dilatable up to maximal 20 mm without significant shortening or loss of stent integrity [10].

The BeGraft stent (Bentley, Hechingen, Germany) is covered with a 200 µm expanded polytetrafluoroethylene (ePTFE) tubing. It is made of Cobalt–Chromium with an open cell design and pre-mounted on a balloon catheter, which goes over a 0.035 inch guidewire. The stent is available in lengths of 19, 29, 39, 49 and 59 mm for diameter sizes of 12 and 14 mm. It is also available in lengths of 19, 29, 38, 48 and 58 mm for diameter sizes of 16 and 18 mm. Finally, it is available in lengths of 27, 37 and 48 mm for diameter sizes of 20, 22, and 24 mm. The 12 and 14 mm diameter stents can be dilated up to maximum diameters of 20 mm, 16 and 18 mm diameter stents to 24 mm, and 20, 22, and 24 mm diameter stents to 30 mm, The required sheath sizes are 9 F (12 mm diameter stent), 11 F (14 and 16 mm), and 14 F (18 mm and above). The nominal and burst pressure for the 12 mm diameter stent is 7 and 10 atm, respectively. The 14 mm diameter stent is 7 and 10 atm, and 16 mm diameter stent is 6 and 9 atm, respectively [11, 12].

### Procedure

**Fig. 1:**
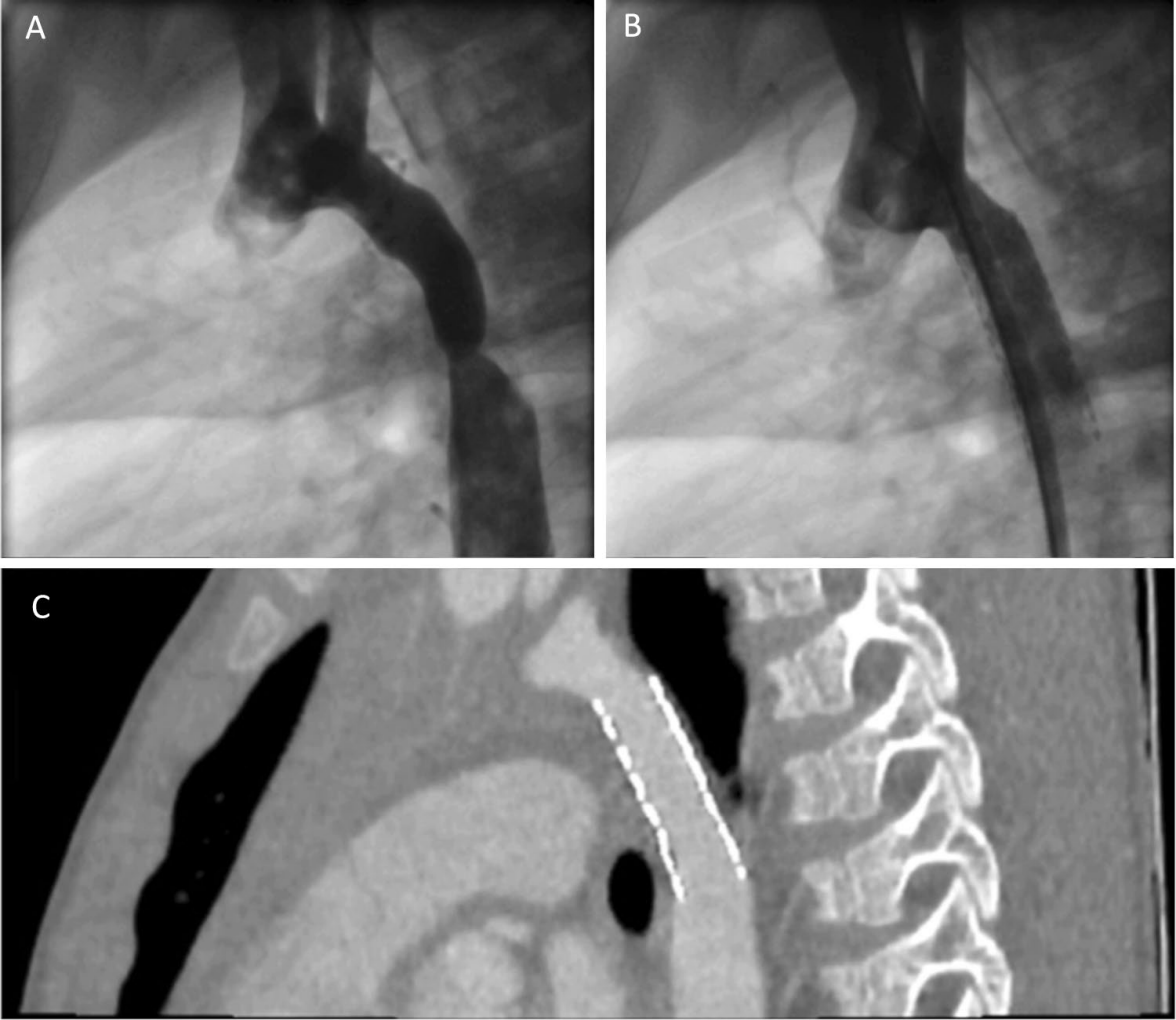
Aortograms and CT of case 17 using Formula 535 stent. **A** Lateral aortogram demonstrating the descending thoracic CoA. **B** Lateral aortogram demonstrating a well-expanded 9 mm × 30 mm stent in position with vessel calibre matching. **C** CT performed 3 months post stent demonstrating no aortic wall complications or stent fractures

**Fig. 2:**
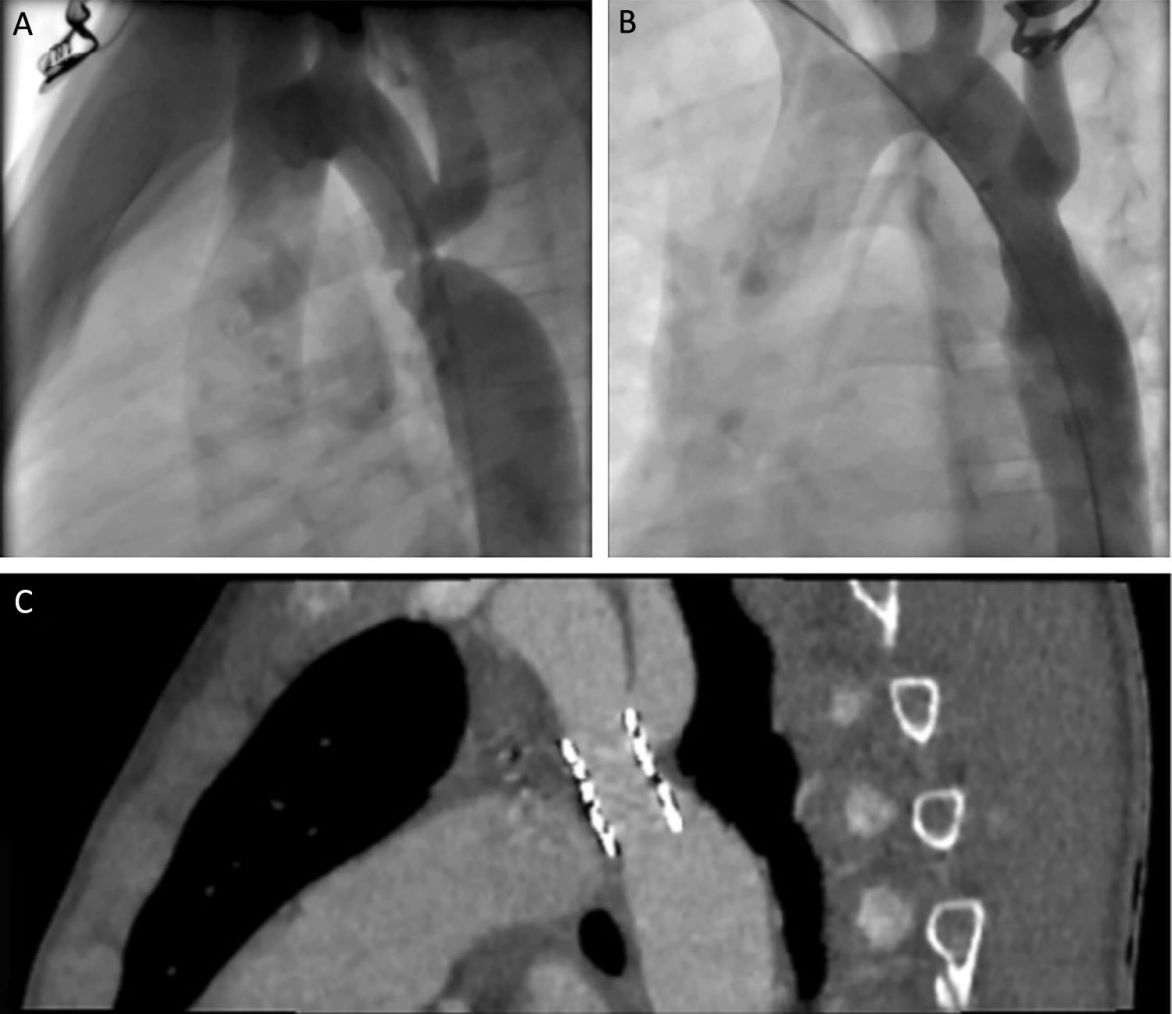
Aortograms and CT of a case using a BeGraft stent. **A** Lateral aortogram shows the left subclavian artery arising posteriorly immediately followed by a severe CoA. **B** Lateral aortogram showing a well-expanded 12 mm × 19 mm stent in position with matching vessel calibre. **C** CT performed 3 months after stent implanting demonstrating no aortic wall complications and preserved stent integrity. Although the stent edge partially covered the origin of the left subclavian artery the patient remained asymptomatic

All stent implantations were performed under general anaesthesia. Valeo™ and Formula^®^ 535 stents were implanted as previously described [7] (Fig. 1). For BeGraft stents (Fig. 2), a 9 F Mullins design 63 cm sheath (Cook, IN, USA) was used to deliver the stent.

## Results

Nineteen patients were included in this study (Table 1). Seven weighed under 20 kg at intervention; all others weighed 20–30 kg. Three types of stents were implanted between 2012 and 2021; 14 Valeo™, 4 Formula^®^ 535, and 1 BeGraft stent. The median age at the time of procedure was 5.1 [4.1–6.4] years and median weight 21.0 kg [17.3–22.3]. One patient had a history of re-coarctation. Thirteen (68%) patients were on anti-hypertensive medications pre-procedure. 7 F sheaths were used in all but one patient, who required a 9 F sheath for a BeGraft stent (Table 2). All patients received follow-up computer tomography (CT) at 3 months. There was no evidence of aortic dissection, aneurysm or pseudoaneurysm formation, stent fracture, deformation or migration or neo-intimal proliferation on CT in any patients. Patients requiring re-intervention showed no evidence of these complications on re-angiogram and there was no stent foreshortening on stent re-dilation (Table 2).

**Table 1:** Summary of patient characteristics, procedural parameters, and follow-up data

	Median [IQR] or *n* (%)
	*N* = 19
Age (year)	5.1 [4.1–6.4]
Weight (kg)	21.0 [17.3–22.3]
Diagnoses	
Coarctation of the aorta Bicuspid aortic valve Ventricular septal defect Double outlet right ventricle Sinus venosus atrial septal defect Left ventricular non-compaction cardiomyopathy Dilated cardiomyopathy Partial anomalous pulmonary venous drainage Bilateral superior vena cava	19 (10) 6 (3) 1 (5) 1 (5) 1 (5) 1 (5) 1 (5) 1 (5) 1 (5)
Transverse arch diameter (mm)	9.2 [8.3–10.0]
Vessel distal to subclavian (mm)	8.9 [8.2–10.0]
Narrowest point of CoA (mm)	3.5 [3.0–4.5]
Diaphragmatic vessel diameter (mm)	10.0 [9.3–12.0]
Mid stent diameter post-intervention (mm)	9.4 [8.9–9.8]^a^
Gradient pre-intervention (mmHg)	35.0 [30.0–43.0]
Gradient post-intervention (mmHg)	5.0 [0–10.0]^a^
Pre-dilation stent length (mm)	26.0 [26.0–30.0]
Maximum balloon diameter (mm)	10.0 [10.0–10.0]
Median sheath size (F)	7.0 [7.0–7.0]
Stent type in initial procedure	
Valeo 10 mm × 26 mm Valeo 9 mm × 26 mm Formula 535 10 mm × 30 mm Formula 535 9 mm × 30 mm BeGraft 12 mm × 19 mm	13 (68) 1 (5) 2 (11) 2 (11) 1 (5)
Follow-up (month)	56.0 [13.0–65.0]
Need for re-intervention	5 (26)
Balloon dilation only in re-intervention	4 (80)
Stenting in re-intervention	1 (20)
Time to re-intervention (month)	40.0 [39.5–52.0]
Anti-hypertensive use pre-intervention	13 (68)
Anti-hypertensive use post-intervention	7 (37)^b^
Long term vascular or cerebral complications	0 (0)

^a^Gradient difference pre-intervention vs. post-intervention and change in diameter post stent were compared using paired student *t*-test which gave *p* < 0.001

^b^Chi-square test of proportions *p* = 0.83

**Table 2:** Summary of patients requiring re-intervention

Baseline diagnosis of cases requiring reintervention	Initial stent type (size in mm)	Time to re-intervention from initial procedure (month)	Reason for re-intervention	Re-intervention procedure(s)	Pre-re-intervention gradient (mmHg)	Post-re-intervention Gradient (mmHg)	Final stent dimensions (mm)	Complications on repeat angiography^a^
CoAo	Valeo 10 × 26	51 87	Keep up with somatic growth Keep up with somatic growth	Stent balloon dilation Stent balloon dilation	18 21	3 10	12 × 26 15 × 26	None None
CoAo	Valeo 10 × 26	39	Keep up with somatic growth	Stent balloon dilation followed by implantation of 14 mm × 39 uncovered CP stent	30	5	14 × 39 (two stent length)	None
CoAo, DORV sub-pulmonary VSD	Valeo 10 × 26	40	Hypertension, keep up with somatic growth	Stent balloon dilation	26	0	13 × 25	None
CoAo	Valeo 10 × 26	40	Aortic flow turbulence, keep up with somatic growth	Stent balloon dilation	1	2	10.5 × 25 11 × 25	None
BAV, CoAo, DCM	Valeo 10 × 26	53	Flow acceleration through stent on echo, keep up with somatic growth	Stent balloon dilation	5	0	11 × 25	None

*BAV* bicuspid aortic valve, *CoAo* coarctation of aorta, *DCM* dilated cardiomyopathy, *DORV* double outlet right ventricle, *Echo* echocardiography, *INT* intervention, *VSD* ventricular septal defect

^a^Complications included stent fracture, foreshortening, neo-intimal proliferation and evidence of pseudoaneurysm on repeat angiography

There was improvement in median coarctation diameter from 3.5 [3.0–4.5] to 9.4 [8.9–9.8] mm, *p* < 0.001; and a reduction in the median pressure gradient across the coarctation from 35.0 [30.0–43.0] to 5.0 [0–10.0] mmHg, *p* < 0.001. There were no short or medium-term complications related to the procedure including the pre-specified cerebral and vascular complications (Table 1).

Follow-up was for a median of 56.0 [13.0–65.0] months. Five (26%) of patients underwent re-intervention; four patients had balloon dilation of the stent, one of which has required who procedures and one had balloon angioplasty followed by immediate stent implant due to a high residual gradient. within a median time frame of 45 [40–53] months. Five (26%) patients were on anti-hypertensive agent(s) post-intervention. Again, none of the aforementioned vascular complications or cerebral complications specified in the methods were present on follow-up.

## Discussion

In the last two decades, stent implantation has emerged as an alternative to surgery in adults and adolescents with CoA and has had good outcomes [3, 5, 13–15]. In small children, stenting is limited by the small number of available stents that can be dilated to adult size, to keep up with somatic growth and the large size of the delivery sheaths, which increase the risk of vascular complications [5, 16–18]. These challenges are being overcome with newer generation stents [7, 19, 20].

Other larger studies have assessed the feasibility of coarctation stenting including one large multicentre study (the COAST) trial. However, the smallest patient in the study was 35 kg [6].

Boe and colleagues demonstrated the feasibility of coarctation stenting in small patients under 20 kg [20]. Their study differs to our due to their use of Palmaz stents, which, unlike the more modern stents used in the present study, are not pre-mounted and require manual mounting and crimping, increasing the risk of error and complications such as “hour-glassing”. In addition, a 9 F or larger sheath was used in nearly 20% of patients in their cohort, where as a 9 F sheath was used in only one case in ours. Whilst Boe et al. were able to demonstrate the feasibility of coarctation stenting in small patients; their complication rate was much higher at around 18%. The present study suggests the superiority of newer generation stents for small patients, including those weighing under 20 kg.

We previously described short term follow-up of coarctation stenting in children under 30 kg using a low-profile balloon expandable [7]. The three stent types that were described could all be introduced via arterial sheaths of 7–9 F in size, which are suitable for small children. In our study, only one patient out of 19 had transiently reduced femoral pulse, which resolved after administration of tissue plasminogen activator. Follow-up vascular ultrasound at 3 weeks showed no complications.

In addition, these low-profile stents can be re-dilated and have the potential to reach adult size. As described previously, the 9- and 10-mm diameter Valeo stent can be dilated up to 20 mm with no loss of stent integrity or length [9]. The BeGraft 12 mm diameter stent up to 20 mm with no significant compromise on the length or integrity of the stent [11]. Bench testing of the Formula 535 stent demonstrated that the 10 mm stents can be dilated up to 20 mm [10]. Use of a short balloon (approximately the same length of the stent) was recommended to prevent “dog-boning” of the stent i.e. preferential expansion of the lateral aspects compared to the middle. They noted that with over-dilation, the Formula stents maintained their length with no shortening, allowing precision placement and minimal protrusion. More recently, we have moved to using these Formula stents where possible and did so in four of the six most recent patients published here due to its’ favourable dilation potential without foreshortening [10].

Indications for stent re-dilation have been varied (Table 2) and are based on clinical and imaging data and a general need to keep with somatic growth. Despite the concerns of the somatic growth spurt in small children and need for re-intervention, in our study, only 5 (26%) of our patient needed re-intervention. Hence, these low-profile stents appear to be safe and effective option to treat CoA in small children.

Thus far, none of the implanted stents have required over-dilation to sizes approximating the maximum achieved dilation on bench testing and no stent fractures have been observed in this cohort. Data from Crystal et al. and Danon et al. demonstrated the feasibility and reliability of intentional stent fracture without “napkin-ring” formation to allow for re-stenting with somatic growth with Formula and Valeo stents [21, 22]. Future publication of longer-term outcomes from our cohort requiring over-dilation will be highly informative.

Of note, the Formula 535 stent is, to date, not FDA approved for use in the United States. The approved Formula 418 stent, which is mounted over a smaller wire, offers an alternative. However, unlike the 14-crown Formula 535 configuration, the 12-crown configuration Formula 418 stent is only available up to 8 mm and can only be post dilated to 14–15 mm before losing it’s radial strength or fracturing [22].

At follow-up, five (26%) of our patients remained on anti-hypertensives over a median follow-up of 56 months despite successful gradient reduction post-intervention and imaging evidence of stent re-stenoses on CT. This is consistent with previous reports that showed 37–57% of patients with CoA having residual hypertension or remain on anti-hypertensives despite correction of anatomical substrate [15, 23, 24]. The pathophysiology is not well understood, however there are reports that suggest the hypertension in CoA is not only contributed by the mechanical obstruction but also several other factors such as arch hypoplasia, abnormal aortic compliance, vascular stiffness and abnormal baroreceptor function resulting in diffuse arteriopathy [25, 26]. Hence, lifelong surveillance is imperative following intervention for those with CoA.

Similar to our previous report [7], this study is limited by its retrospective single-centre design and small patient sample.

## Conclusions

Use of small profile balloon expandable stents is a feasible and effective long-term treatment option in children under 30 kg with CoA, with very low risk of complications. Repeated balloon dilations to match somatic growth, is to be expected but did not have to be repeated frequently in our series with only 5 out of 19 patients requiring re-intervention during the period of follow-up.
